# ${\mathcal{P}}{\mathcal{T}}$-Symmetric Effective Model for Nonequilibrium Phase Transitions in a Dissipative Fermionic Mott Insulator Chain

**DOI:** 10.1038/s41598-020-64222-x

**Published:** 2020-04-29

**Authors:** V. Tripathi, V. M. Vinokur

**Affiliations:** 1 Department of Theoretical Physics, Tata Institute of Fundamental Research, Homi Bhabha Road, Navy Nagar, Mumbai, 400005 India; 2 Materials Science Division, Argonne National Laboratory, 9700 S. Cass Ave, Argonne, IL 60439 USA; 3 Consortium for Advanced Science and Engineering (CASE) University of Chicago, 5801 S Ellis Ave, Chicago, IL 60637 USA

**Keywords:** Phase transitions and critical phenomena, Phase transitions and critical phenomena

## Abstract

Nonequilibrium phase transitions in open dissipative systems can be described as instabilities in the spectra and wavefunctions of effective non-Hermitian Hamiltonians invariant under simultaneous parity ($\boldsymbol{\mathcal{P}}$) and time-reversal ($\boldsymbol{\mathcal{T}}$) transformations. The degree of non-Hermiticity reflects the strength of the external drive and dissipation, and the transition is described as a loss of the $\boldsymbol{\mathcal{P}}\boldsymbol{\mathcal{T}}$ symmetry of the solutions corresponding to stationary low-drive dynamics. This approach has been successfully applied to spin, superconducting, and Mott insulator systems. However, the microscopic foundations for the employed phenomenological models are currently lacking. Here we propose a microscopic mechanism leading to the $\boldsymbol{\mathcal{P}}\boldsymbol{\mathcal{T}}$-symmetric effective model in the context of the nonequilibrium Mott transition in a dissipative Hubbard chain. Our model comprises a half-filled fermionic Hubbard chain subject to a constant electric field. The dissipation is introduced via the electron-phonon coupling. We obtain the explicit expressions for the non-Hermitian parameter in terms of the electron-phonon coupling strength and driving field. Analyzing the implications of microscopic model, we find a re-entrant Mott insulator with the increasing electric field for phonon density of states that increases slower than the square of the energy (such as in one or two dimensions), or varies non-monotonously with energy.

## Introduction

There has been a recent breakthrough in the physics of non-equilibrium systems extending the Hamiltonian approach onto the open dissipative out-of-equilibrium systems by using non-Hermitian Hamiltonians with the non-Hermiticity determined by the external drive and dissipation^1,2^, see also^3,4^ for a review. In this approach an energy gain and loss balanced nonequilibrium system is described by non-Hermitian Hamiltonians possessing the parity-time (${\mathcal{P}}{\mathcal{T}}$) symmetry, hence real energy spectrum. Increasing the degree of non-Hermiticity, associated with both the dissipation and driving force, results in the loss of the ${\mathcal{P}}{\mathcal{T}}$ symmetry of the eigenstates. At the ${\mathcal{P}}{\mathcal{T}}$ symmetry-breaking transition the eigenvalues of the non-Hermitian Hamiltonian acquire finite imaginary components^5^ implying that the system transits from stationary to non-stationary dynamics. This approach has been employed in quantum optics and photonic systems^6–11^, microwave cavities^12^, superfluid dynamics^13,14^, vortex depinning^15,16^, and remarkably enabled the quantitative description of the dynamic Mott transition^17–20^, resistive transitions in current-biased superconducting wires^1,21^, and dynamic phase transitions in spin systems^22,23^.

In all the above developments, except^21^, where an imaginary term in the Hamiltonian for a superconducting wire appeared in the linear approximation with respect to the applied electric field, the non-Hermiticity have been introduced on a phenomenological level, whereas the microscopic origin of the imaginary drive has remained an open question. However, the successful description of such a rich diversity of physical systems, indicates a possible generality in underlying microscopic mechanisms behind the non-Hermiticity. An example of such a generality was set in an early paper by Obukhov^24^ where a field theory formulation of the directed percolation problem yielded an imaginary vector potential driving the directed percolation transition transition. This theme was also picked up by Van Lien and Shklovskii^25^ who reduced the electronic hopping conductivity in strongly-disordered semiconductors subject to a strong electric field to a problem of the directed percolation. Inspired by these considerations, we demonstrate an emergence of the effective constant imaginary component of the vector potential in a half-filled fermionic dissipative Hubbard chain establishing thus a microscopic foundation for phenomenological ${\mathcal{P}}{\mathcal{T}}$-symmetric descriptions of the nonequilibrium phase transitions in electronic systems.

We investigate a half-filled fermionic Hubbard chain with fermions experiencing a uniform constant electric field. Dissipation is introduced via the electron-phonon coupling. Elimination of the phonon degrees of freedom using a *T*-matrix approach results in a non-Hermitian ${\mathcal{P}}{\mathcal{T}}$-symmetric model for the fermions, a Hubbard model with an imaginary vector potential. We derive the explicit expressions for the non-Hermitian parameters in terms of the electron-phonon coupling strength and the driving field. The model is integrable and yields the electric field dependence of the critical value of the vector potential at which the ${\mathcal{P}}{\mathcal{T}}$-symmetry breaking transition occurs. We identify an unusual metal to Mott insulator transition driven by the *increasing* electric field in certain physical situations such as where the phonon density of states increases slower than the square of the phonon energy, such as in one or two dimensions, or has a non-monotonous dependence on the energy.

## The model

We start with the one-dimensional fermion lattice subjected to a constant electric field and coupled to an external phonon bath. Transforming to a Wannier-Stark basis, we show that the system behaves as an insulator in the absence of electron-phonon coupling, and that this coupling facilitates hopping between different localized Wannier-Stark orbitals. Next, eliminating the phonon degrees of freedom, we show how an effective ${\mathcal{P}}{\mathcal{T}}$-symmetric microscopic Hamiltonian emerges on account of unequal probabilities for phonon emission and absorption processes.

Let us first consider noninteracting spinless fermions (the interacting case will be taken up below) hopping over a one-dimensional lattice:

1 $${H}_{{\rm{el}}}^{(0)}=-\,t\,\sum _{\langle ij\rangle }\,[{c}_{i}^{{\rm{\dagger }}}{c}_{j}+{\rm{h}}.\,{\rm{c}}.]+\sum _{i}\,{\epsilon }_{i}{n}_{i}.$$

Here ${\epsilon }_{j}=eEj$ is the potential energy due to the constant electric field $E,$ and ${n}_{i}={c}_{i}^{{\rm{\dagger }}}{c}_{i}$ is the fermion number operator at the site labeled $i\mathrm{}.$ This Hamiltonian is diagonalized by the transformation

2 $${f}_{n}=\sum _{i}\,{J}_{i-n}(2t/eE){c}_{i}\,,$$

where ${J}_{n}$ are the Bessel functions. This yields a discrete spectrum in a form of the Wannier-Stark ladder with energies ${E}_{n}=n(eE)$, and $n$ being integer. The wavefunctions corresponding to energies ${E}_{n}$ are localized and center around the respective sites $n$ with the spatial extent of order of $L=2t/eE$. Since the excitation spectrum is discrete and off-diagonal terms connecting the localized Wannier-Stark orbitals are not present in the Hamiltonian, no dc charge transport is possible. To enable dc transport, we introduce inelastic scattering processes (e.g. due to an electron-phonon interaction) that allow a fermion to relax its energy during the hop between two localized Wannier-Stark states. We consider a weak coupling of fermions with the phonon bosonic bath,

3 $${H}_{{\rm{el}}-{\rm{ph}}}=-\,\sum _{i}\,\sum _{q}\,{n}_{i}[F(q){e}^{iqn}{b}_{q}+{\rm{h}}.{\rm{c}}\mathrm{.],}$$

where ${b}_{q}$ annihilates a phonon with momentum $q,$ and $F(q)$ describes the nature of electron-phonon coupling. For our purposes, it suffices to regard $F(q)\equiv \lambda$ as a constant. We model the phonon bath by harmonic oscillators,

4 $${H}_{{\rm{ph}}}=\sum _{q}\,{\omega }_{q}{b}_{q}^{{\rm{\dagger }}}{b}_{q}\,,$$

where the phonon distribution function ${n}_{{\rm{ph}}}(\epsilon )$ need not be the equilibrium Bose distribution. The total Hamiltonian is thus $H={H}_{{\rm{el}}}^{\mathrm{(0)}}+{H}_{{\rm{ph}}}+{H}_{{\rm{el}}-{\rm{ph}}}$. As this approach is easily generalized to fermions endowed with spins, we will omit the spin index unless necessary. To see how electron-phonon coupling enables fermion hopping, we transform to the Wannier-Stark creation and annihilation operators *f*^†^, *f* instead of *c*^†^ and *c* making use of Eq. (2) and the orthogonality relation ${\sum }_{n}\,{J}_{n-m}(x){J}_{n-m{\prime} }(x)={\delta }_{mm{\prime} }$; whence^26^

5 $$\begin{array}{rcl}H & = & \sum _{n}\,{\epsilon }_{n}{f}_{n}^{{\rm{\dagger }}}{f}_{n}+\sum _{q}\,{\omega }_{q}{b}_{q}^{{\rm{\dagger }}}{b}_{q}\\  &  & +\,\sum _{n,\ell ,q}\,[{F}_{\ell }(q){e}^{iqn}{b}_{q}+{\rm{h}}.{\rm{c}}.]{f}_{n+\ell }^{{\rm{\dagger }}}{f}_{n},\end{array}$$

where

6 $${F}_{\ell }(q)={i}^{\ell }\lambda {J}_{\ell }(4t\,\sin (q/2)/eE){e}^{iq\ell /2}.$$

It is evident that the electron-phonon coupling term that is not simultaneously diagonalized by the transformation in Eq. (2), facilitates the phonon-assisted hopping between the pairs of the localized Wanner-Stark orbitals ${f}_{n}$. The $\ell =0$ term adds/removes phonons at the given orbital and results in polaronic corrections to the Wannier-Stark energy eigenvalues. For simplicity, we specifically consider the case of strong electric fields such that $4t/eE\ll 1$, and the Wannier-Stark orbitals are highly localized. Since $4t/eE\ll 1$, we have approximately ${F}_{0}(q)\approx \lambda$. The electron hopping between different, $\ell \ne 0$, Wannier-Stark states is always accompanied by the emission or absorption of a phonon. For large fields, the arguments of the Bessel functions in Eq. (5) are small and decrease rapidly with order $\ell ,$ since ${J}_{\ell }(x)\propto {(x/2)}^{|\ell |}/|\ell |!$ for $x=4t\,\sin (q/2)/eE\ll \sqrt{|\ell |+1}$. This allows us to restrict to phonon-assisted tunneling between neighboring Wannier-Stark orbitals, $\ell =0,\pm \,1$, and ignore hops between more distant states. We replace the Bessel function in the $\ell =\pm \,1$ terms by the small argument approximation, i.e. ${F}_{1}(q)\approx i\lambda {e}^{iq/2}(2t\,\sin (q/2)/eE)$ and ${F}_{-1}(q)\approx i\lambda {e}^{-iq/2}(2t\,\sin (q/2)/eE)$. The decrease of the phonon-assisted hopping matrix element with increasing field is a consequence of shrinking of the Wannier-Stark orbitals with electric field. The bottleneck in the fermion transport are the quantities ${F}_{\pm 1}(q)\sim \lambda t/eE\ll {F}_{0}(q)\simeq \lambda$. The rate of phonon equilibration with the bath, governed by ${F}_{0}(q),$ is thus large compared to the rate with which they are emitted/absorbed during phonon-assisted hopping. We thus consider only incoherent exchange of phonons with the bath, and ignore polaronic processes involving coherent emission/absorption of phonons during hopping. We argue now that this approximation of inelastic scattering directly leads to our desired ${\mathcal{P}}{\mathcal{T}}$-symmetric effective model. In what follows, we regard the electron-phonon coupling term in the Hamiltonian as an interaction, ${H}_{I},$ and the bare Hamiltonian comprising the Wannier-Stark ladder ${\sum }_{n}\,{\epsilon }_{n}{f}_{n}^{{\rm{\dagger }}}{f}_{n}$ and the phonon term ${\sum }_{q}\,{\omega }_{q}{b}_{q}^{{\rm{\dagger }}}{b}_{q}$.

The electron-phonon coupling terms involve either phonon creation or annihilation operators. One could integrate out the massless phonons and obtain an effective fermion-only model, but this procedure will result in an action with non-local in time interactions, and our goal is to obtain an effective fermionic Hamiltonian for the description of nonequilibrium steady states. So, instead, we view the electron-phonon coupling as a source of inelastic scattering involving an emission or absorption of a phonon. The corresponding scattering rates for hopping down or against the field gradient must be different since the probabilities for emission and absorption of phonons respectively involve the factors $\mathrm{(1}+{n}_{{\rm{ph}}})$ and ${n}_{{\rm{ph}}}\mathrm{}.$ To see that, we replace the phonon operators ${b}_{q}$ and ${b}_{q}^{{\rm{\dagger }}}$ by the corresponding elements of the $T$-matrix. For the phonon emission ${T}_{q0}=\langle T{e}^{-i\int {H}_{I}dt}{b}_{q}^{{\rm{\dagger }}}\rangle$ and for phonon absorption ${T}_{0q}=\langle T\,{e}^{-i\int {H}_{I}dt}{b}_{q}\rangle$. Here the averaging is performed over a phonon bath and $T\,{e}^{-i\int {H}_{I}dt}$ is the $S$-matrix. The phonon emission rate is proportional to $1+{n}_{{\rm{ph}}}({\omega }_{q})$, while the absorption rate is proportional to ${n}_{{\rm{ph}}}({\omega }_{q})$, where ${\omega }_{q}$ is the energy of the phonon involved and ${n}_{{\rm{ph}}}({\omega }_{q})$ their distribution function. Thus, for the phonon absorption process we have

7 $$\begin{array}{rcl}{T}_{0q}(n+\ell ,n) & \approx  & \langle {(1-i\int {H}_{I}(t)dt)}_{n+\ell ,n}{b}_{q}\rangle \\  & = & -i2\pi {F}_{\ell }^{\ast }(q){e}^{-iqn}\delta ({\omega }_{q}+{E}_{n}-{E}_{n+\ell }){n}_{{\rm{ph}}}({\omega }_{q}),\end{array}$$

where the $\delta$-function imposes conservation of energy, and we used $\langle {b}_{q}^{{\rm{\dagger }}}{b}_{q}\rangle ={n}_{{\rm{ph}}}({\omega }_{q})$. The total energy at the site is the sum of the electrostatic energy of the fermion and the energy due to the boson modes. Since in our regime of interest, phonon-assisted hopping is dominated by the emission and absorption of a single phonon carrying the energy $eE$, the phonons at any site equilibrate with the bath much faster than their creation or annihilation during which the phonon-assisted hopping occurs. Consequently, near the steady state transport conditions, the phonon density is uniform and static, and the energy difference of the successive Wannier-Stark sites is $eE$ even in the presence of the electron-phonon coupling^26^. Since ${E}_{n+\ell }-{E}_{n}\approx \ell (eE),$ the energy conservation in Eq. (7) is possible only for $\ell \ge 0$. As discussed above we retain only $\ell =0,1$. Specifically, the $\ell =1$ gives us the $T$-matrix for inelastic hopping to the nearest Wannier-Stark orbital against the field gradient, accompanied by absorption of a phonon. We now replace the boson creation and annihilation operators in the interaction part of the Hamiltonian, Eq. (5), with corresponding $T$-matrix elements. The leading order contribution to the inter-orbital hopping process comes from the $\ell =\pm \,1$. We substitute the $T$-matrix elements in the interaction part of Eq. (5), taking only dominant terms (i.e., $\ell =0,\pm \,1$). Then the matrix elements for the forward and backward hopping with respect to the field gradient are simply the (inelastic) Raman scattering elements,

8 $$\begin{array}{rcl}{t}_{{\rm{f}}} & \sim  & {(\lambda t/eE)}^{2}{\rho }_{{\rm{ph}}}(eE\mathrm{)(1}+{n}_{{\rm{ph}}}(eE)),\\ {t}_{{\rm{b}}} & \sim  & {(\lambda t/eE)}^{2}{\rho }_{{\rm{ph}}}(eE){n}_{{\rm{ph}}}(eE),\end{array}$$

where $\lambda$ is the electron-phonon coupling constant. Since $|{t}_{{\rm{f}}}|\ne |{t}_{{\rm{b}}}|$, a non-Hermitian tight-binding model arises with the constant imaginary vector potential:

$${H}_{{\rm{eff}}}=\sum _{n}{E}_{n}f_{n}^{{\rm{\dagger }}}{f}_{n}-\sum _{n}\,[{t}_{{\rm{f}}}f_{n+1}^{{\rm{\dagger }}}{f}_{n}+{t}_{{\rm{b}}}f_{n}^{{\rm{\dagger }}}{f}_{n+1}],$$

where ${E}_{n}=-\,neE+{\rm{const}}.$ We now eliminate the potential gradient by a gauge transformation, so that

9 $$\begin{array}{rcl}{H}_{{\rm{eff}}} & = & -\,\sum _{n}\,[{t}_{{\rm{f}}}{e}^{-ieE\tau }{f}_{n+1}^{{\rm{\dagger }}}{f}_{n}+{t}_{{\rm{b}}}{e}^{ieE\tau }{f}_{n}^{{\rm{\dagger }}}{f}_{n+1}]\\  & \equiv  & -{t}_{{\rm{h}}}\,\sum _{n}\,[{e}^{-ieE\tau +\Psi }{f}_{n+1}^{{\rm{\dagger }}}{f}_{n}+{e}^{ieE\tau -\Psi }{f}_{n}^{\dagger }{f}_{n+1}],\end{array}$$

where ${t}_{f(b)}={t}_{h}\,\exp$[±Ψ] and $\tau$ represents time.

Introducing correlations in the form of electron repulsion between neighboring sites, one obtains a spinless Hubbard model with asymmetric hopping. For spinful electrons, we obtain a ${\mathcal{P}}{\mathcal{T}}$-symmetric non-Hermitian Hubbard model,

10 $$\begin{array}{rcl}H & = & -{t}_{{\rm{h}}}\,\sum _{n,\sigma }\,[{e}^{-ieE\tau +\Psi (E)}{f}_{n+\mathrm{1,}\sigma }^{{\rm{\dagger }}}{f}_{n\sigma }+{e}^{ieE\tau -\Psi (E)}{f}_{n\sigma }^{{\rm{\dagger }}}{f}_{n+\mathrm{1,}\sigma }]\\  &  & +\,U\,\sum _{i}\,{n}_{i\uparrow }{n}_{i\downarrow }\mathrm{}.\end{array}$$

For a thermal distribution of phonons, we have Ψ$(E)=eE\beta /2$ and ${t}_{{\rm{h}}}(E)\sim \frac{{(\lambda t/eE)}^{2}{\rho }_{{\rm{ph}}}(E)}{2\,\sinh (eE\beta /2)}$.

In the regime of not very small fields which we have studied above, the Wannier-Stark orbitals are highly localized at the individual lattice sites, and local correlations in the original model correspond to local correlations in the Wannier-Stark basis. Note that even in our strong electric field regime, the Coulomb correlation scale $U$ could still be much larger than $t\mathrm{}.$ For small fields, the Wannier-Stark orbitals extend over multiple sites and the approach presented here becomes complicated to implement.

## Results and discussion

The derivation of the ${\mathcal{P}}{\mathcal{T}}$-symmetric microscopic model in Eq. (10) is the primary achievement of ths paper. While the model has been studied previously^2,27^ in the context of nonequilibrium phase transitions in Mott insulator chains, it was motivated phenomenologically, and the suggested form of the non-Hermitian contribution (proportional to the current operator), lacked a microscopic justification.

The presence of the non-Hermitian perturbation dramatically affects the physical properties of the Mott insulator. Earlier studies^2,27^ have shown that when the parameter Ψ controlling the non-Hermiticity increases to a certain threshold value

11 $${\Psi }_{c}(E)\approx {\sinh }^{-1}\left[\frac{U}{4{t}_{{\rm{h}}}(E)}\right],$$

the system described by the Hamiltonian (10) experiences a phase transition from a Mott insulator to a bad metal state. This is in stark contrast with the behavior of the half-filled Hermitian fermionic Hubbard chain that does not exhibit a phase transition under the applied drive^28^ but in agreement with Keldysh field-theory based analyses of driven dissipative Mott insulator systems^29^. Approaching the transition, the Mott gap Δ collapses, showing the critical behavior $\Delta \sim \sqrt{{\Psi }_{c}-\Psi }\propto \sqrt{{E}_{c}-E}$^2^, that has subsequently been confirmed by experiments^19^.

The electric field dependence of the non-Hermitian parameter in Eq. (10) that arises from the asymmetry between the forward and backward tunneling elements (${t}_{{\rm{f}}}$ and ${t}_{{\rm{b}}}$, see Eq. (8)) has important implications for our understanding of the nonequilibrium response of driven Mott insulators at strong fields. Consider the hopping elements in Eq. (8). For acoustic phonons, assuming a phonon density of states of the form ${\rho }_{{\rm{ph}}}(eE)\sim {(eE)}^{d-1},$ ($d$ being the dimensionality of the phonon bath in local thermal equilibrium with the electrons), we find that the asymmetry of hopping, ${t}_{{\rm{f}}}-{t}_{{\rm{b}}}\sim {(\lambda t)}^{2}{(eE)}^{d-3},$ decreases with increasing electric field for the case of $d=1,2$. This can also happen for a bath where the phonon density of states has a non-monotonous dependence on the energy. The implication is that for sufficiently strong electric fields, the value of Ψ (*E*) may fall below the minimum value Ψ_*c*_ (*E*) required for the transition resulting in the possibility of an unusual re-entry from the bad metal to Mott insulator phase. Physically, this re-entry occurs when the phonons are unable to provide the energy to effect the inelastic hopping between the Wannier-Stark levels whose separation increases with electric field. To our understanding, such a bad metal to Mott insulator phase driven by increasing electric field has not been studied in the literature.

Consider for example a thermal distribution of phonons. The expression for the field dependent Ψ_*c*_ assumes the form

12 $${\Psi }_{c}(E)\approx {\sinh }^{-1}\left[\frac{{(eE)}^{2}U}{2{(t\lambda )}^{2}{\rho }_{{\rm{ph}}}(E)}\,\sinh (eE\beta /2)\right].$$

Evidently, if ${\rho }_{{\rm{ph}}}(E)$ increases slower than ${E}^{2},$ the right hand side of Eq. (12) increases faster than Ψ $(E)=eE\beta /2$, ultimately resulting in a Mott insulator phase at higher fields.

Our analysis was done for the specific case of weak intersite hopping (or strong electric fields). In the opposite limit of strong intersite hopping or weak electric fields, the Wannier-Stark orbitals are spatially large, overlapping significantly in real space, and the electron-phonon coupling generates very nonlocal and comparable hopping elements, ${F}_{\ell }\sim \lambda {J}_{\ell }(4D|\,\sin (q/2)|/eE)\sim \lambda \sqrt{eE/2\pi D}$, between overlapping Wannier-Stark orbitals. Interestingly, the phonon-assisted hopping elements between neighboring Wannier-Stark orbitals have a nonmonotonous dependence on the electric field. The hopping matrix elements between non-overlapping Wannier-Stark orbitals separated by distances $\ell$ greatly exceeding the size of a Wannier-Stark orbital $D/eE$ are much smaller, ${F}_{\ell }\sim \lambda {(2D/eE)}^{\ell }/\ell !$. Despite the complicated nature of the weak field regime in the Wannier-Stark basis, it is nevertheless clear that the hopping between different orbitals would vanish unless the electron-phonon coupling $\lambda$ and the electric field are both nonzero. Elimination of dynamical phonon degrees of freedom using the approximation of replacing with static averages followed in this paper for the strong electric field case will once again lead to asymmetric scattering rates along and against the field direction (because of different probabilities associated with phonon emission and absorption), leading in principle to a non-Hermitian effective model. Correlations introduce another, possibly more serious, level of difficulty in the treatment of the weak field regime. The Hubbard interaction, that is local in real space, becomes very nonlocal in the Wannier-Stark basis for small electric fields, although it is evident that significant correlations exist only between electrons in spatially overlapping Wannier-Stark orbitals. This suggests coarse-graining to a lattice scale comparable to the Wannier-Stark orbital size as a possible way forward. Clearly, further studies are needed to derive an effective ${\mathcal{P}}{\mathcal{T}}$-symmetric description for dissipative Hubbard chains subjected to weak electric fields.

An open question remains concerning the transient response of these systems to the sudden switching of the nonequilibrium drive. The expected initial response is the Bloch-like oscillations, which have to cross over into the eventual steady-state dc current, the response that is absent in the non-dissipative counterpart^28,30^. For driven dissipative Mott systems, nonequilibrium field theoretical approaches have been developed, primarily based on the Keldysh dynamical mean-field theory^31–33^ for the Hubbard model, or more recently, the Keldysh generalization of the Ambegaokar-Eckern-Schön (AES) rotor model for normal granular metals^29^ indeed demonstrating the envisioned behavior. It would be very interesting to investigate whether the nonequilibrium insulator-to-metal transitions in these Keldysh-based models are the true phase transitions or crossovers, and in the former case, how do the critical exponents of the field driven nonequilibrium Mott transition in the Keldysh approach compare with those obtained from the ${\mathcal{P}}{\mathcal{T}}$-symmetric Hubbard model^2^.

## Conclusion

To conclude, we demonstrated a microscopic origin of non-Hermitian ${\mathcal{P}}{\mathcal{T}}$-symmetric Hamiltonians that had been phenomenologically introduced earlier for the description of the nonequilibrium phase transitions in fermionic Hubbard chains at half filling. The parameter associated with non-Hermiticity was found to comprise a combination of the driving field and the parameter controlling the strength of relaxation processes, and could be interpreted as an effective constant and purely imaginary component of the vector potential. Formulating the problem in terms of an explicit ${\mathcal{P}}{\mathcal{T}}$-symmetric model enabled us to identify the nonequilibrium phase transition as a ${\mathcal{P}}{\mathcal{T}}$-symmetry breaking phenomenon. The explicit expressions for the electric field dependencies of the non-Hermitian parameter Ψ and the effective phonon-mediated hopping ${T}_{{\rm{h}}}$ between the Wannier-Stark states reavealed the possibility of the unusual field-driven bad metal-to-Mott insulator phase transition. We showed that such a transition is possible in situations where the phonon density of states increases slower than ${E}^{2},$ such as in one and two dimensions, or decreases with the increasing energy.

In deriving the ${\mathcal{P}}{\mathcal{T}}$-symmetric Hamiltonian, we approximated the full phonon dynamics with the static distributions, which assumes a fast local and incoherent equilibration of the phonons as compared to the phonon-assisted electron hopping. The opposite situation where both, electron and phonon, distributions are dynamically evolving in a strongly correlated system is a challenging problem which is beyond the purview of this work. Our analysis was limited to the regime of sufficiently strong electric fields such that the Wannier-Stark orbitals do not extend over multiple sites. This was needed to preserve the local nature of the Coulomb correlations even in the Wannier-Stark basis. The problem of a ${\mathcal{P}}{\mathcal{T}}$-symmetric description in the opposite limit of weak electric fields where Wannier-Stark orbitals extend over multiple sites will be a subject of the forthcoming publication.

## Data Availability

The authors declare that all relevant data supporting the findings of this study are available within the article.
